# Extradigital Glomus Tumor at the Elbow

**DOI:** 10.5334/jbsr.3755

**Published:** 2024-11-12

**Authors:** Lucas Van Houtven, Dieter Peeters, Filip M. Vanhoenacker

**Affiliations:** 1 AZ Sint‑Maarten, Department of Radiology, Mechelen, University Antwerp, Belgium; 2 AZ Sint‑Maarten, Department of Pathology, Mechelen, Faculty of Medicine and Health Sciences, University Antwerp, Belgium; 3 AZ Sint‑Maarten Mechelen, Department of Radiology and University (Hospital) Antwerp/Ghent, Belgium

**Keywords:** extradigital glomus tumor, MRI, ultrasound, color doppler

## Abstract

*Teaching point:* Extradigital glomus tumor should be considered in the differential diagnosis of a highly vascular solid lesion that is painful on palpation.

## Case Presentation

A 43‑year‑old male presented with a longstanding history of sharp, shooting pain at the medial aspect of the left elbow radiating to the distal forearm and exacerbated by palpation.

**Figure 1.**
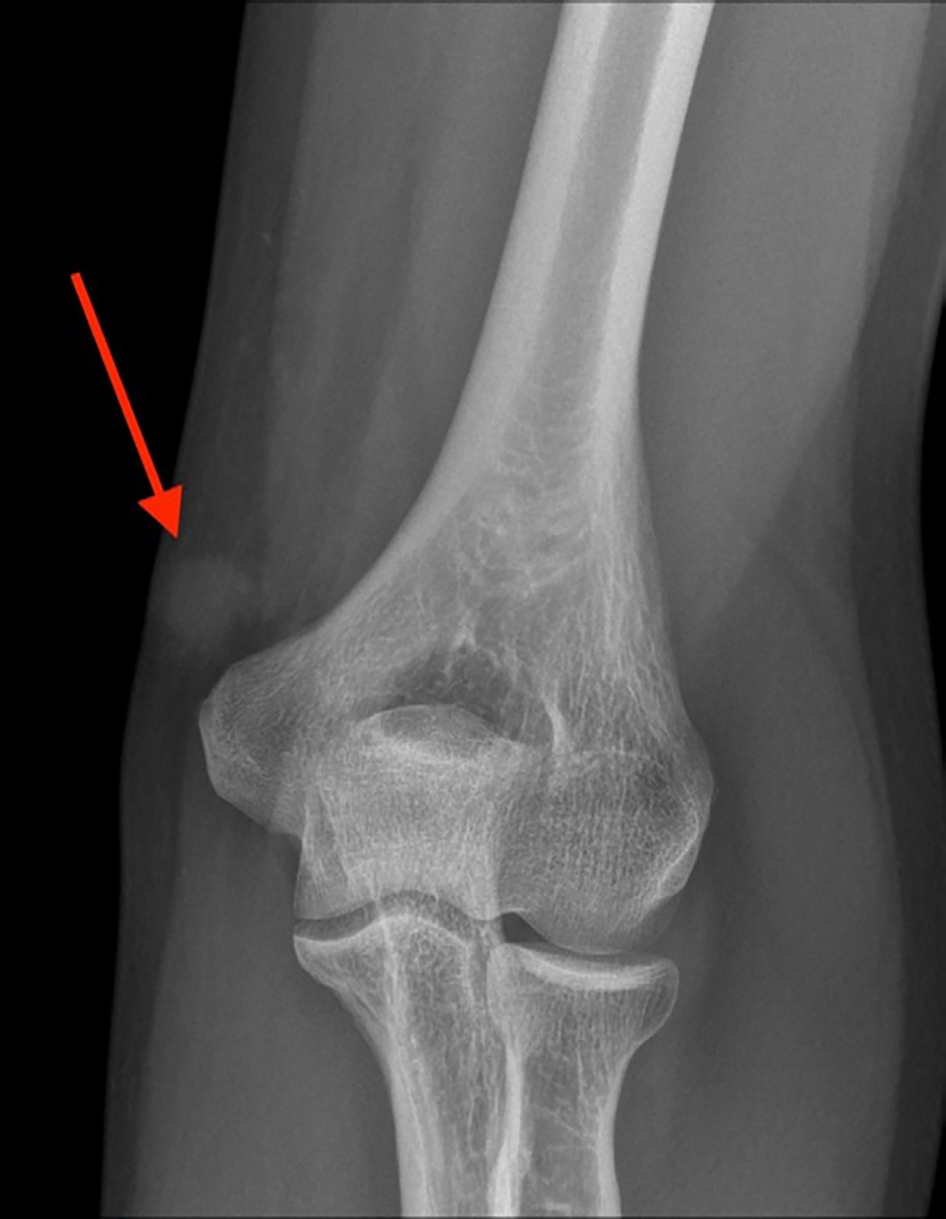
Conventional radiography of the left elbow reveals a nodular soft tissue opacity proximal to the medial epicondyle (arrow).

Conventional radiography (CR) (Figure 1) revealed a nodular soft tissue opacity proximal to the medial epicondyle. Due to pronounced pain on palpation, initial ultrasound (US) was not possible.

**Figure 2 A‑C.**
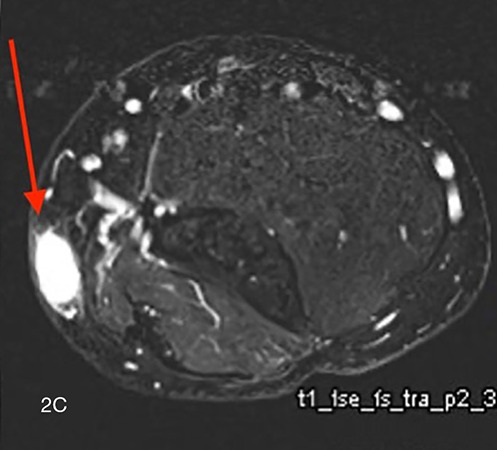
Magnetic Resonance Imaging shows an oval‑shaped, well‑defined nodule in the subcutaneous tissue. **A.** The lesion appears isointense to muscle on T1‑weighted images (arrow) and **B.** is hyperintense on fatsuppressed T2‑weighted images (arrow). **C.** After intravenous administration of gadolinium contrast, there is vivid, homogeneous enhancement of the lesion (arrow), in keeping with a vascular tumor.

Magnetic resonance imaging (MRI) showed an oval‑shaped, well‑defined nodule in the subcutaneous tissue. The lesion appeared isointense to muscle on T1‑weighted images (Figure 2A, arrow) and hyperintense on fat‑suppressed T2‑weighted images (Figure 2B, arrow). After intravenous administration of gadolinium contrast, there was vivid homogeneous enhancement of the lesion (Figure 2C, arrow), suggesting a vascular tumor.

**Figure:**
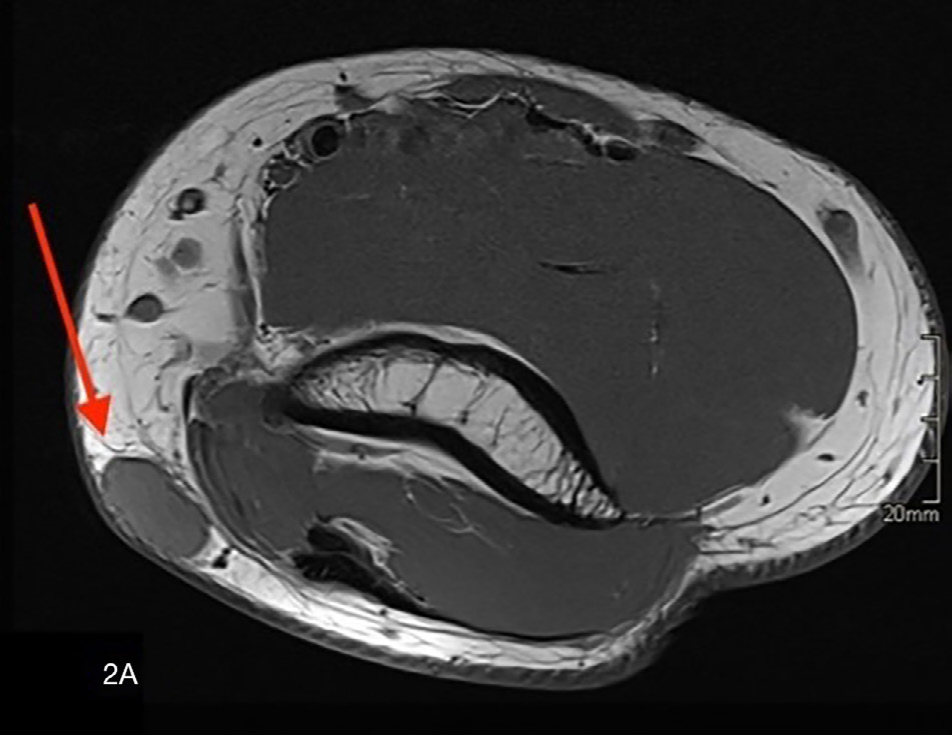


**Figure:**
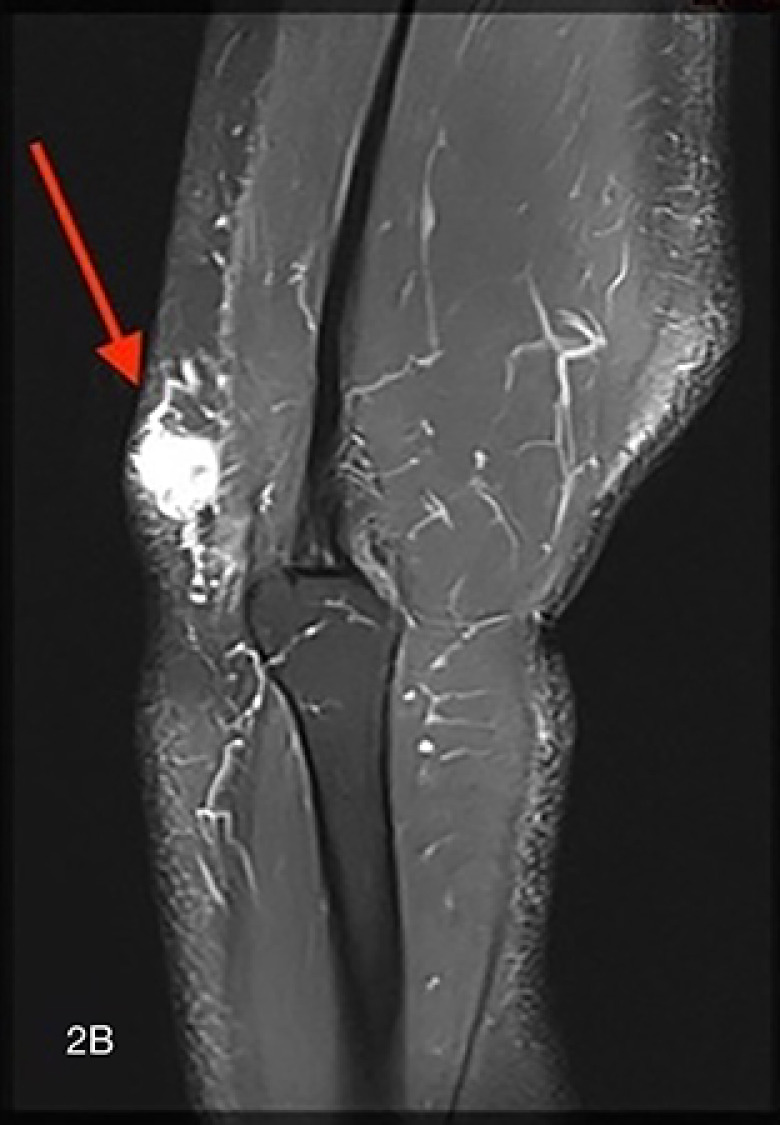


Targeted US confirmed a subcutaneous, well‑delineated, noncompressible hypoechoic nodule (Figure 3A) with retro‑acoustic enhancement and high vascularity at color Doppler (Figure 3B).

**Figure 3 A.**
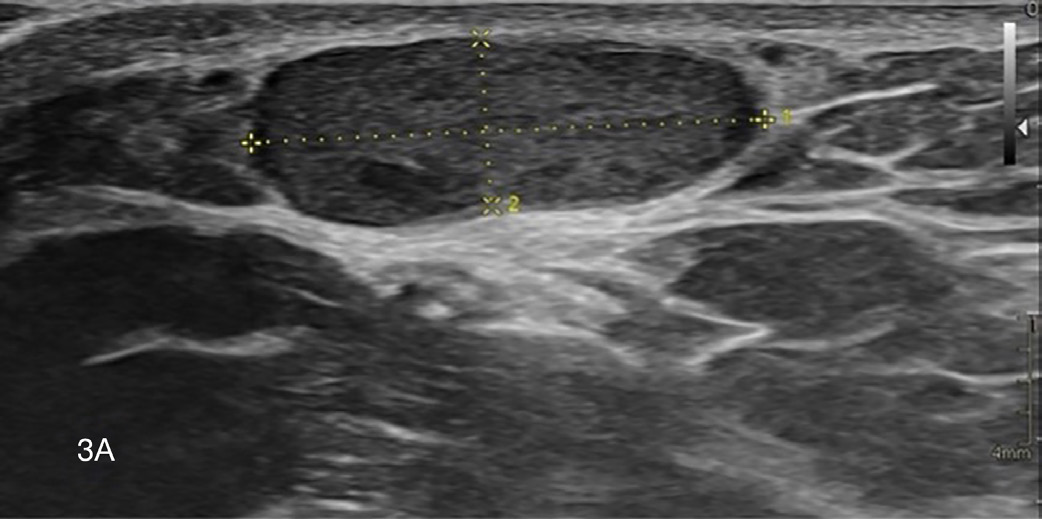
Ultrasound shows a subcutaneous hypoechoic nodule.

**Figure 3 B.**
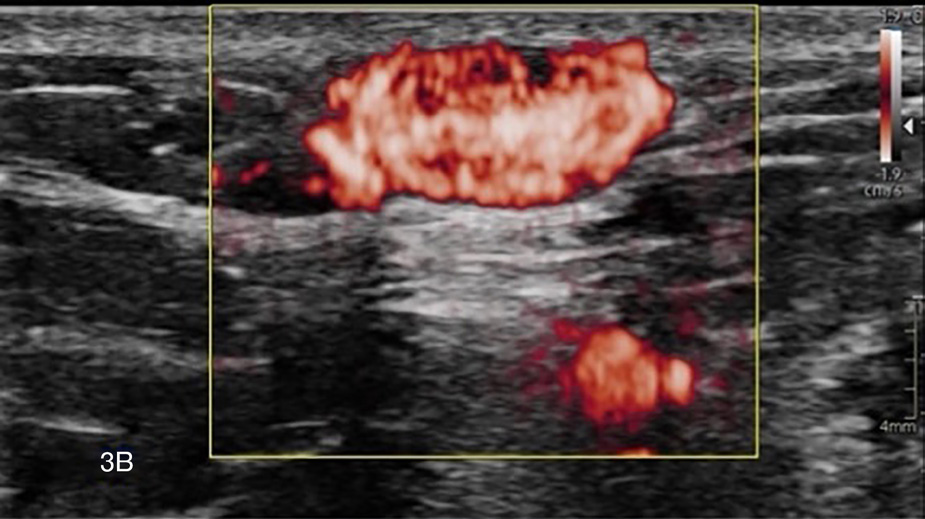
There is retro‑acoustic enhancement and high vascularization on color Doppler.

The lesion was resected and histopathology revealed a well‑circumscribed lesion containing branching capillaries lined by endothelial cells surrounded by trabeculae of uniform glomus cells in a background of hyalinized and myxoid stroma.

Immunohistochemistry was positive for CD31 in the capillaries and for smooth muscle actin and vimentin in the glomus cells. The final diagnosis of an extradigital glomus tumor was obtained.

## Comment

Glomus tumors are rare benign vascular tumors originating from the glomus body, which affects blood pressure and thermoregulation. They are typically found in the digits but can occur in other body parts, causing intense localized pain. Unlike digital glomus tumors, extradigital tumors do not cause increased sensitivity to cold, potentially leading to a delayed diagnosis.

Conventional X‑ray usually does not contribute to the diagnosis but may show a nonspecific subcutaneous soft tissue opacity. On US, the lesion is hypoechoic with high vascularization on color Doppler. MRI is particularly valuable for defining the tumor’s size, location, and vascular nature, showing an isointense signal to muscle on T1‑weighted images and a hyperintense signal on T2‑weighted images with marked homogeneous contrast enhancement [1]. Combining imaging findings with the clinical presentation of pinpoint pressure pain is key to suggesting the diagnosis.

Histopathological confirmation is still required for a final diagnosis. Surgical resection is curative, providing immediate pain relief and minimizing the risk of recurrence.

## References

[r1] Zanjani LO, Sadeghi R, Saffar N, Rezaei MH, Chavoshi M, Zareinezhad H. An unusual case of chest wall glomus tumor presenting with axillary pain: A case report and literature review. Eur J Med Res. 2021;26(1):49. DOI: 10.1186/s40001-021-00514-4. 34034818 PMC8146208

